# Ground Contact Force and Moment Estimation for Human–Exoskeleton Systems Using Dynamic Decoupled Coordinate System and Minimum Energy Hypothesis

**DOI:** 10.3390/biomimetics8080558

**Published:** 2023-11-21

**Authors:** Hongwu Li, Haotian Ju, Junchen Liu, Ziqi Wang, Qinghua Zhang, Xianglong Li, Yi Huang, Tianjiao Zheng, Jie Zhao, Yanhe Zhu

**Affiliations:** State Key Laboratory of Robotics and System, Harbin Institute of Technology, Harbin 150001, China; lhwhit@126.com (H.L.); juhaotian_hit@foxmail.com (H.J.); liujc_hit@163.com (J.L.); zq_wang01@outlook.com (Z.W.); 19b908069@stu.hit.edu.cn (Q.Z.); 18b908105@stu.hit.edu.cn (X.L.); yihuang_hit@163.com (Y.H.); zhengtj@hit.edu.cn (T.Z.); jzhao@hit.edu.cn (J.Z.)

**Keywords:** ground contact force and moment estimation, exoskeleton, dynamic decoupled coordinate system, minimum energy hypothesis

## Abstract

Estimating the contact forces and moments (CFMs) between exoskeletons’ feet and the ground is a prerequisite for calculating exoskeletons’ joint moments. However, comfortable, portable, and high-precision force sensors for CFM detection are difficult to design and manufacture. In addition, there are many unknown CFM components (six force components and six moment components in the double-support phase). These reasons make it challenging to estimate CFMs precisely. In this paper, we propose a novel method for estimating these CFMs based on a proposed dynamic decoupled coordinate system (DDCS) and the minimum energy hypothesis. By decomposing these CFMs into a DDCS, the number of unknowns can be significantly reduced from twelve to two. Meanwhile, the minimum energy hypothesis provides a relatively reliable target for optimizing the remaining two unknown variables. We verify the accuracy of this method using a public data set about human walking. The validation shows that the proposed method is capable of estimating CFMs. This study provides a practical way to estimate the CFMs under the soles, which contributes to reducing the research and development costs of exoskeletons by avoiding the need for expensive plantar sensors. The sensor-free approach also reduces the dependence on high-precision, portable, and comfortable CFM detection sensors, which are usually difficult to design.

## 1. Introduction

The development of exoskeletons makes it possible for humans have more endurance and be stronger during locomotion. Learning-based methods, like human-in-the-loop (HIP), have the potential to reduce wearers’ energy costs for typical gait conditions, e.g., walking and running [1,2,3,4,5]. The joint moment parameters (peak torque, peak time, fall time, rise time, etc.) during a complete gait cycle can be optimized using these optimization frameworks. However, a minimum gait cycle unit (usually two steps during walking and running) is necessary for these optimization frameworks [6,7,8], which means these methods do not work for random locomotion. Moreover, the performance of these data-driven methods depends heavily on the diversity of the data in the data set. To enable the exoskeleton to adapt to richer action scenes, a method that is not reliant on a normalized periodic gait or rich data sets is still necessary for exoskeleton control.

Dynamics-modeling-based methods are usually more robust as well as more adaptable and have been widely used for biped robots to calculate the joint moments during locomotion [9,10]. They compensate for the insufficiencies of learning-based methods. Generally, dynamics-modeling-based methods calculate the moments of exoskeletons’ joints according to the contact forces and moments (CFMs) as well as inverse dynamics [11,12,13,14]. CFMs can be detected using well-designed high-precision force sensors [15,16,17,18,19]. However, comfortable, portable, and high-precision force sensors for measuring these CFMs are difficult to design and manufacture [20,21,22,23]. In addition, the shape, hardness of the ground surface, and the contact state between the feet and the groundill also affect the accuracy of the measurement results [24,25].

To solve this problem, many researchers have studied sensor-free methods to estimate CFMs. These methods regard the human and exoskeleton as a multi-rigid-body system and solve CFMs as unknowns according to D’Alembert’s principle. The key is how to calculate the 12 unknown CFM components (including 6 force and 6 moment components) using only the 6 provided force balance equations. In many studies, to reduce the number of unknown CFM components, the moment components under the sole are partially or completely ignored, as in [26,27,28]. Although these studies provide methods to solve the CFMs, the accuracy of the CFMs is reduced. In addition to reducing the unknowns, increasing the additional restraints is feasible for this problem. To provide additional restraints, some researchers suggested establishing objective functions and additional complementary energy equations [29]. However, the optimization process is still difficult due to the six additional unknown variables. In recent years, a musculoskeletal-modeling-based approach has been developed to predict external kinetics from kinematic data [9,30,31]. This kind of method requires a high-precision model of human muscle, bones, and soft tissue; it also requires the knowledge of the contact stiffness between the foot and the ground. These parameters are difficult to estimate in practical situations. Authors [10] completed the estimation by setting a spline curve about the COP locus. However, it is not clear whether the spline curve is also suitable when the gait changes. To summarize, the mismatch between the number of unknowns and constraints remains a key challenge in current CFM estimation methods.

In view of these issues, in this paper, we propose a novel method for estimating CFMs based on a dynamic decoupled coordinate system (DDCS) and the minimum energy hypothesis. The former transforms the CFMs into five solvable components and two parameters to be optimized, while the latter provides an efficient and fast solution method to solve the two parameters. The inverse dynamics matrix for solving the CFMs cannot be solved due to singularity, which is a problem that the DDCS resolves, considerably lowering the number of parameters that need to be optimized. We verify the accuracy of this method with a public data set about human walking. The validation shows that the proposed method is able to effectively estimate the CFMs.

## 2. Coordinate Systems for Exoskeletons

In order to solve the CFMs under the feet of human–exoskeleton systems, it is necessary to establish a coordinate system to describe the distribution of the CFMs. According to the origin position of the coordinate system and the orientation of the coordinate axes, there are two main types of coordinate systems in the existing research:

### 2.1. Coordinate Systems with Origins on the Extreme of the Supporting Leg

**Figure 1:**
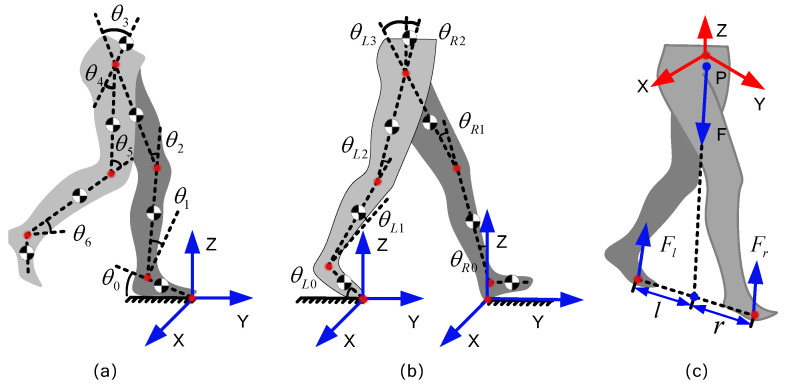
Existing dynamic modeling methods. (**a**) Coordinate systems with origins on the extreme of the supporting leg. (**b**) Coordinate systems in double-supporting phases. (**c**) Estimation of the CFMs in double-supporting phases.

One common way to establish a coordinate system for exoskeletons is by setting the origin of the coordinate system at the extreme of the supporting leg [28,32,33,34,35]. This method establishes different coordinate systems during different phases of locomotion. In single-supportphases, exoskeletons are modeled as a multi-DOF serial link mechanism in the sagittal plane [28], as shown in Figure 1a. In double-support phases, both feet are nearly flat on the ground. The exoskeletons are modeled as two planar multi-DOF serial link mechanisms that are connected along their uppermost link [28], as shown in Figure 1b. The origins of the coordinate systems are set up at the two ends of the serial links (the ankle joints). The contributions of the CFMS under each foot are chosen as functions of the location of the torso center of mass relative to the locations of the ankles, as shown in Figure 1c. This method actually simplifies the spatial dynamics problem as a plane problem. In this way, there are only 2 vertical contact forces under each foot left, and the other 10 components (6 moments and 4 forces components) are ignored.

### 2.2. Floating-Based Coordinate System

Another common way to establish a coordinate system is by setting the origin of the coordinate system on the torso of humans [12,24]. The exoskeleton is regarded as a floating-base multi-link structure. For this method, the coordinate origin is always located at the location of the torso center of mass and does not have to change constantly, as shown in Figure 1c. However, it adopts a similar method of calculating the CFMs to the method in Section 2.1. The CFMs under the feet are still acquired from force sensors or calculated using approximate linearization functions. Therefore, the measurement deviation of the sensing system and the errors caused by the linearization still affects the exoskeleton control performance.

## 3. Singularity of Force System Equilibrium Equation

Both ways of establishing coordinate systems in Section 2 simplify the CFMs as planar force systems in double-support phases. In fact, even though we establish more force balance equations in a space force system, we still cannot solve more unknown CFM components. We analyze the reason in this section. In short, the problem is primarily caused by the coupling of the CFM components.

**Figure 2:**
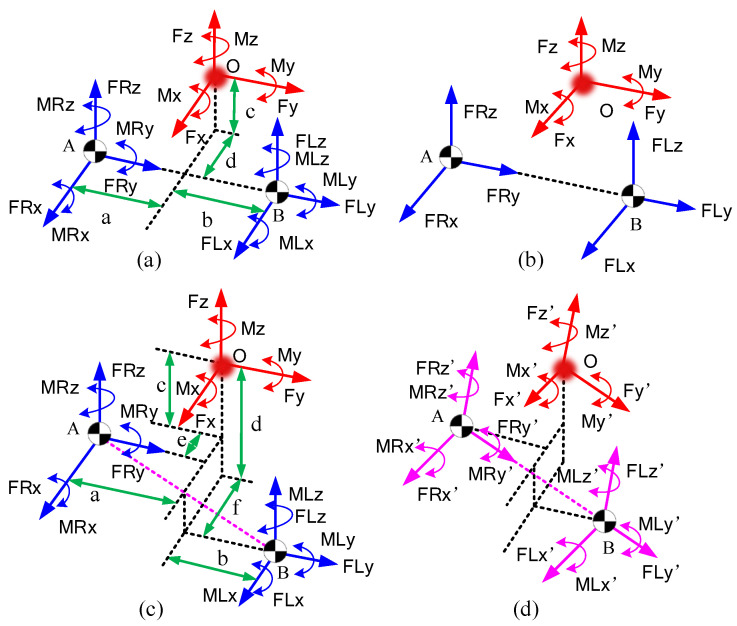
Simplifying the CFMs. (**a**) The CFMs when line AB is parallel to the *Y*-axis. (**b**) The CFMs when moment components are ignored. (**c**) The CFMs when line AB is not parallel to the *Y*-axis. (**d**) The dynamic decoupled coordinate system to make sure AB is parallel to the *Y*-axis in general states.

In actual engineering, since it is intractable to measure the force distribution on the sole of the foot, the distributed force under each foot is reduced to a concentrated force system acting on one point on the sole of the foot in most related studies. Figure 2 describes these CFMs in a floating-based coordinate system. Let us start with a special case, as shown in Figure 2a, where the points *A* and *B* represent the acting points of the two concentrated force systems, *O*-XYZ is the coordinate system of the exoskeleton system, and the forces FRY and FLY are collinear in this special condition. The resultant effect of FRY and FLY is certain once the sum of FLY and FRy is certain, no matter what change happens on one of the two components. In other words, if we only focus on the resultant effect on the whole body, FRY and FLY have infinite combinations of solutions. Not only the force components FLy and FRy but also the moment components MLx and MRx, MLy and MRy, and MLz and MRz have the same issues. Even if we ignore the moment components to reduce the 6 unknowns as in most existing work, as shown in Figure 2b, FLy and FRy still cannot be calculated. As long as the sum of FLy and FRy is certain, there are myriads of solutions.

The couplings between these force components lead to a result where part of the CFMs cannot be solved in the existing space force system. What is even more troubling is that this coupling exists not only in some special cases but in all postures. We further analyze the coupling situation and principle between CFM components in the following sections.

Next, we mathematically prove the conclusion that even if the 6 moment components of the CFMs are ignored to simplify the estimation, the 6 force components are still unsolvable. Furthermore, we prove the problem exists not only in some special situations but also in all human postures.

First, we start from the special human posture in Figure 2b, the line AB is parallel to OY. If the 6 moment components of the CFMs are ignored to simplify the estimation, we can establish a dynamic equation in the coordinate system *O*-XYZ as follows:

(1) $$\left\{\begin{array}{c} FL_{X}+FR_{X}=F_{X}\\ FL_{Y}+FR_{Y}=F_{Y}\\ FL_{Z}+FR_{Z}=F_{Z}\\ cFL_{Y}+cFR_{Y}+bFL_{Z}-aFR_{Z}=M_{X}\\ cFL_{X}+cFR_{X}-dFL_{Z}-dFR_{Z}=M_{Y}\\ -bFL_{X}+aFR_{X}+dFL_{Y}+dFR_{Y}=M_{Z} \end{array}\right.$$

We write Equation (1) in matrix form as follows:

(2) Ax=F

where

(3) $$A=\left[\begin{array}{cccccc} 1 & 0 & 0 & 1 & 0 & 0\\ 0 & 1 & 0 & 0 & 1 & 0\\ 0 & 0 & 1 & 0 & 0 & 1\\ 0 & c & b & 0 & c & -a\\ c & 0 & -d & c & 0 & -d\\ -b & d & 0 & a & d & 0 \end{array}\right]$$

(4) $$\begin{array}{c} x={\left[FL_{X},FL_{Y},FL_{Z},FR_{X},FR_{Y},FR_{Z}\right]}^{T}\\ F={\left[F_{X},F_{Y},F_{Z},M_{X},M_{Y},M_{Z}\right]}^{T} \end{array}$$

Thus, we can calculate the CFMs *x* using:

(5) $$x=A^{-1}F$$

However, from Equation (3), we obtain:

(6) rank(A)=5

Thus, the matrix *A* is singular and irreversible, meaning that Equation (1) cannot be solved and there are many solutions.

Fortunately, when the line between points *A* and *B* is parallel to one axis, as shown in Figure 2a, although FLy and FRy still cannot be calculated, the sum of the two components and the other four force components are solvable. For example, we let:

(7) $$F_{YSUM}=FL_{Y}+FR_{Y}$$

Equation (1) becomes:

(8) $$\left\{\begin{array}{c} FL_{X}+FR_{X}=F_{X}\\ F_{YSUM}=F_{Y}\\ FL_{Z}+FR_{Z}=F_{Z}\\ cF_{YSUM}+bFL_{Z}-aFR_{Z}=M_{X}\\ -bFL_{X}+aFR_{X}+dF_{YSUM}=M_{Z} \end{array}\right.$$

Thus,

(9) $$A=\left|\begin{array}{ccccc} 1 & 0 & 1 & 0 & 0\\ 0 & 0 & 0 & 0 & 1\\ 0 & 1 & 0 & 1 & 0\\ 0 & b & 0 & -a & c\\ -b & 0 & a & 0 & d \end{array}\right|$$

(10) $$\begin{array}{c} x={\left[FL_{X},FL_{Z},FR_{X},FR_{Z},F_{YSUM}\right]}^{T}\\ F={\left[F_{X},F_{Y},F_{Z},M_{X},M_{Z}\right]}^{T} \end{array}$$

(11) rank(A)=5

In Equation (8), since the equation is a linear combination of the other equations, we can reduce the moment balance equation along OY. Because rank(A) is equal to the number of the unknowns, Equation (8) can be solved. Thus, we calculate $FL_{X}$, $FL_{Z}$, $FR_{X}$, $FR_{Z}$, and $F_{YSUM}$. This helps to greatly reduce the number of unknowns, so that it is easier to establish an objective function to acquire an optimized set of solutions.

The reason that Equation (8) can be solved is that line AB is parallel to axes OY, so we can easily find the relationship between $FL_{Y}$, $FR_{Y}$, and $F_{Y}$. However, when line AB is not parallel to any axis, as shown in Figure 2c, it is difficult to directly establish the relationship between $FL_{Y}$, $FR_{Y}$, and $F_{Y}$, as in Equation (8); so, this method will no longer be used.

However, rank(A) is still smaller than the number of unknowns in this situation. As shown in Figure 2d, to analyze this further, we can transmit the CFMs into a new coordinate system to make sure $OY^{\prime }$ coincides with line AB. The fundamental theorem of linear algebra proves that homogeneous transformations do not affect the rank of a matrix. Thus, if we establish a dynamic equation in Figure 2c,d, where *A* and $A^{\prime }$ are the coefficient matrices of the two dynamic equations, respectively, the rank of *A* is equal to that of $A^{\prime }$. In Section 3, we show that the rank of the coefficient matrix *A* is 5 when AB is collinear with the axes. Thus,

(12) $$rank\left(A\right)=rank\left(A^{\prime }\right)=5$$

Therefore, there is a myriad of solutions about $FR_{Y}$ and $F_{Y}$.

To summarize, regardless of whether line AB is collinear with one of the coordinate axes, the dynamic Equation (1) cannot solve the 6 unknown force components (the 6 neglected torque components are not included). However, when line AB is collinear with the coordinate axis, although the coupled force components still cannot be calculated, the sum of the two components and the other four force components can be calculated. This greatly reduces the number of unknowns, thus reducing the difficulty of estimating the pressure on the sole of the foot.

## 4. Method

### 4.1. The DDCS

According to the analysis in Section 2, we draw the following important conclusions:

(1) Because of the coupling between CFM components, it is impossible to solve the three-dimensional space force system in traditional coordinate systems. Too many unknown CFMs create great difficulties for the solution of dynamic equations as well as the estimation of CFMs.
(2) When AB coincides with the coordinate axis, the spatial force system is solvable by merging the collinear CFM components. In this way, the number of unknowns is tremendously reduced.
(3) Only in special cases in traditional coordinate systems can the collinear CFM components be merged.

To reduce the unknown CFMs, in this study, we established a DDCS. In the DDCS, the 12 unknown CFM components can be transformed into five solvable components and two parameters to be optimized, thus making it easier to establish an objective function to acquire an optimized set of solutions. This greatly reduces the difficulty of estimating the CFMs under the feet.

#### 4.1.1. Reducing the Moment Components Based on the Centers of Pressure (COP) of the Feet

**Figure 3:**
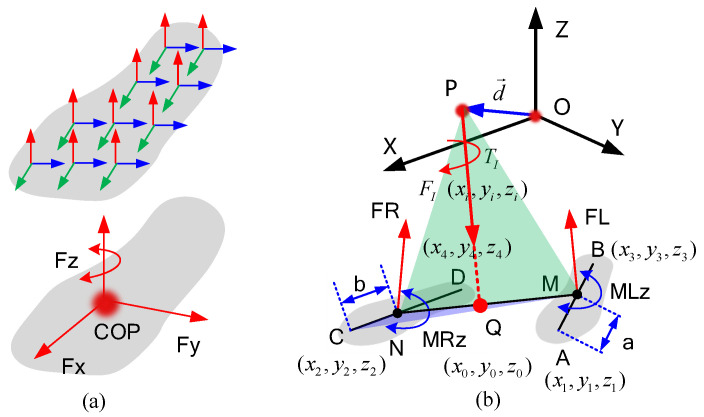
Reducing the unknowns based on the COPs under the feet. (**a**) Simplification of the distribution of force on the sole of the foot. (**b**) Simplified CFMs.

In this section, we provide a method to reduce the unknown CFM moments by finding the COPs of each foot. As shown in Figure 3a, there are perpendicular and tangential distributed forces under the feet of the exoskeleton. According to the principles of traditional statics, we can find a point in the foot sole plane such that all the distributed forces perpendicular to the foot sole plane have a moment of zero about this point ( point COP in Figure 3a). If we simplify the distributed force to the COP point, the CFMs of each foot can be reduced to 3 orthogonal forces and a moment perpendicular to the soles of the feet, as shown in Figure 3b.

Furthermore, the moment is caused by the tangential force under the foot, which has relatively little effect on human movement. This moment is usually generated by the internal rotation of the legs, which usually occurs when humans make large turns. In many studies, this moment component was neglected to reduce the number of unknowns. For example, in [29], only the moments perpendicular to the sagittal plane were considered, and the other moment components were neglected. In [9], the moment components parallel to the ground were eliminated by moving the GRFs to the COPs, and the moments perpendicular to the ground were neglected. There are also some other works that neglected the moment components perpendicular to the ground [28,36].

In this paper, we also neglect the two components. Finally, there are only 6 force components acting on the two COPs of the feet, and the unknown CFM components are reduced from 12 to 8 (6 forces, AM, and CN).

However, the COPs change during the locomotion of human bodies. According to D’Alembert’s principle, by applying inertial forces to rigid objects, dynamic problems can be transformed into static problems. By simplifying the inertia forces acting on the whole body, we can finally determine the inertia force and the inertia moment in the same direction. The $T_{I}$ and $F_{I}$ in Figure 3b represent the simplified inertia force and moment of inertia, respectively. In the single-supporting phases, there is only one foot connecting with the ground; thus, the projection of $F_{I}$ must be inside the contour of the supporting foot. In other words, when the projection is inside the contour of one foot, the subpoint is just the COP of the supporting foot. In this condition, the CFMs of the supporting foot are equal and opposite to $F_{I}$, and the CFMs under the other foot are zero. In double-supporting phases, if we use lines AB and CD to represent the soles of the two feet and use *M* and *N* to represent the COPs of each foot, as shown in Figure 3b, the CFM of each foot can be simplified as a resultant force acting on the COP (FR and FL), as analyzed above. According to the balance principle of the force system, the points *M* and *N* and the inertia force $F_{I}$ must be on the same plane. Thus, the inertia force $F_{I}$ and the line MN must intersect (assuming the intersection of two lines is *Q*). This means as long as the position of one COP (equal to the length of AM) is ensured, the position of the other COP (length of CN) is also ensured. There is only one unknown variable to represent the position of COPs *M* and *N*. Therefore, the 12 unknown CFM components can be reduced to 1 unknown *a* (the length of AM) and 6 force components, so there are 7 unknowns in total.

During the process above, we approximated the human foot as a line segment without considering the width of the foot, and we also ignored the torque generated by the tangential force under the foot soles when the person turns. In fact, the effect of these factors on the calculation results is small.

#### 4.1.2. Method to Establish the DDCS

**Figure 4:**
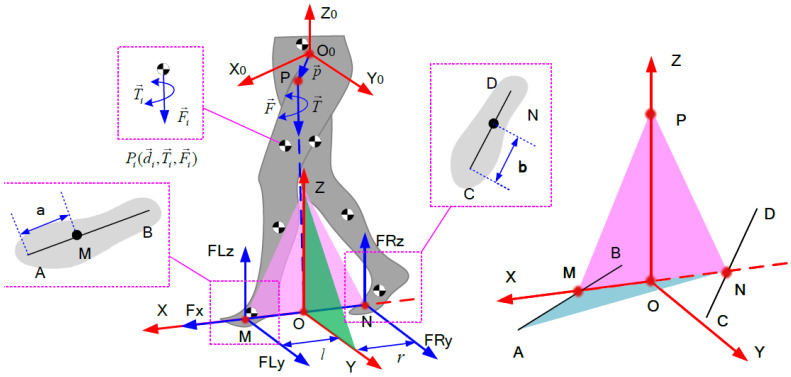
Process of establishing DDCS and the simplified process for CFMs based on the DDCS.

By simplifying the distributed force at the bottom of the foot to the COP, we reduce the number of unknowns to seven. Even so, the number of unknowns is still too large to optimize. To further reduce the number of unknowns, we propose a DDCS. The steps for establishing the coordinate system are as follows:

(1) First, we choose a point near the center of mass of the human body as the origin of the coordinate system ($O_{0}$−$X_{0}Y_{0}Z_{0}$) on the back of the human body.
(2) We simplify the inertial forces and moments distributed in the human body, resulting in a pair of forces and moments in the same direction ($\vec{F}$ and $\vec{T}$ in Figure 4). The specific calculation process is described in Equations (13)–(15). First, the resultant force and the resultant moment at point $O_{0}$ can be calculated as
  (13) $$\begin{array}{c} \vec{F}=\sum_{i=1}^{n}{\vec{F}}_{Ii}\\ {\vec{T}}_{I}=\sum_{i=1}^{n}{\vec{F}}_{Ii}\times {\vec{d}}_{Ii}+\sum_{i=1}^{n}{\vec{T}}_{Ii} \end{array}$$
  where ${\vec{d}}_{i}$ is the vector that points to the center of mass of the body part *i*; ${\vec{T}}_{Ii}$ and ${\vec{F}}_{Ii}$ are the inertia force and moment of the corresponding body part. Next, the simplified inertia force and moment can be calculated as:
  (14) $$\vec{T}=\frac{\vec{F}\times {\vec{T}}_{I}}{\left|\vec{F}|\right|{\vec{T}}_{I}|}\vec{F}$$
  (15) $$\vec{p}=\frac{\vec{F}\times \vec{T}}{\left|\vec{F}|\right|\vec{F}|}$$
  where $\vec{F}$ and $\vec{T}$ are the simplified inertia force and moment. $\vec{p}$ is the offset of the simplified resultant and moment lines with respect to the coordinate origin $O_{0}$.
(3) Determining the coordinates of COP positions *M* and *N* in the coordinate system $O_{0}$–$X_{0}Y_{0}Z_{0}$ (Here, we assume that *a* and *b* are known). According to the previous analysis, line MN and line OP must intersect, and we assume the intersection is point *O*, as shown in Figure 4. Since the points *A*, *O*, *B*, and *N* are in the same plane, and the points *P*, *M*, *O*, and *N* are also in the same plane, we have
  (16) $$\vec{AM}\times \vec{BN}•\vec{MN}=0$$
  (17) $$\vec{PM}\times \vec{PN}•\vec{F}=0$$
  According to Equations (16) and (17), we can finally establish the relationship between *a* and *b* as follows:
  (18) $$b=-\frac{\vec{PM}\times \vec{F}•\vec{PC}}{\vec{PM}\times \vec{F}•\vec{CD}}\left|\vec{CD}\right|$$
  Let θ represent the joint angles of the human body; thus, the position of points *P*, *C*, and *D* is the function of θ. Since position *M* is ensured according to *a*, according to Equation (18), *b* can be expressed as a function of *a* and θ as follows:
  (19) b=f(a,θ)
  (20) $$\theta =(\theta_{1},\theta_{2},...\theta_{i})$$
  Therefore, when θ is certain, *b* can be represented by *a*, and there is only one unknown variable *a* to locate the two COPs of the foot. The position of *O* is also ensures by (16) and (17) as follows:
  (21) $$\vec{OO_{0}}=\vec{O_{0}P}+\frac{\vec{NA}\times \vec{AB}•\left(\vec{O_{0}M}-\vec{O_{0}P}\right)•\vec{F}}{\vec{NA}\times \vec{AB}•\vec{F}}$$
(4) Finally, we define point *O* as the origin of the new coordinate system, and let *X*-axis coincide with line OM. Let the *Y*-axis ge through point *O* and perpendicular to plane PNM. Thus, the *Z*-axis is also confirmed according to the *X*-axis and *Z*-axis. In this way, the DDCS is finally established. The direction vector of *X*-axis, *Y*-axis, and *Z*-axis can be calculated as:
  (22) $$\vec{OX}=\frac{\vec{OM}}{\left|OM\right|}$$
  (23) $$\vec{OY}=\frac{\vec{OM}\times \vec{OP}}{\left|\vec{OM}\times \vec{OP}\right|}$$
  (24) $$\vec{OZ}=\vec{OX}\times \vec{OY}$$

The point of the DDCS is that the *X*-axis always coincides with the connecting of two COPs. Under this condition, the rank of the coefficient matrix of equation set (8) is 5, so that the value of the CFMs is solvable using Equation (8). Finally, we rewrite the equation set (8) as follows:

(25) $$\left\{\begin{array}{c} F_{X}=F_{XD}\\ FL_{Y}+FR_{Y}=F_{YD}\\ FL_{Z}+FR_{Z}=F_{ZD}\\ -lFL_{Z}+rFR_{Z}=M_{YD}\\ lFL_{Y}-rFR_{Y}=M_{ZD} \end{array}\right.$$

where *l* and *r* are shown in Figure 4. According to Equation (19), they are both functions of *a*. $F_{X}D$, $F_{Y}D$, $F_{Z}D$, $M_{Y}D$, and $M_{Z}D$ are expressions of inertial force and the moment of inertia. In DDCS coordinates, it can be calculated using Equation (27).

(26) $$F_{D}={\left[F_{XD},F_{YD},F_{ZD},M_{YD},M_{ZD}\right]}^{T}$$

(27) $$F_{D}=RF$$

where R∈$R^{6\times 6}$ is the transformation matrix from DDCS to Cartesian coordinates. The equation set (25) is solvable; thus, we can obtain the CFMs in DDCS using:

(28) $$x=A^{-1}F_{D}$$

(29) $$x={\left[F_{X},FL_{Y},FL_{Z},FR_{Y},FR_{Z}\right]}^{T}$$

where *A* is the coefficient matrix of equation set (25). It is obvious that the matrix *A* is full rank, so Equation (28) is solvable.

(30) $$A=\left[\begin{array}{ccccc} 1 & 0 & 0 & 0 & 0\\ 0 & 1 & 0 & 1 & 0\\ 0 & 0 & 1 & 0 & 1\\ 0 & 0 & -l & 0 & r\\ 0 & l & 0 & -r & 0 \end{array}\right]$$

When setting up the dynamics of the exoskeleton as a whole system, we can use $F_{X}$ to replace the component of the CFMs along the *X*-axis. However, when calculating the joint moments of each leg, we need to divide the force between the two COP points. Therefore, we supplement the relationship as follows:

(31) $$\begin{array}{c} FL_{X}=kF_{X}\\ FR_{X}=\left(1-k\right)F_{X} \end{array}$$

*l* and *r* in matrix *A* represent the distance between *O* and the COPs *M* and *N* in Figure 4; thus, they are the functions of *a*, there are finally two unknowns *a* and *k* as total. Thus,

(32) $$F_{CFM}=f_{ak}\left(a,k\right)$$

(33) $$F_{CFM}={\left[FL_{X},FR_{X},FL_{Y},FL_{Z},FR_{Y},FR_{Z}\right]}^{T}$$

where f(a,k) is a linear function, and $F_{CFM}$ is the CFMs in the DDCS coordinate system.

To summarize, the space force system is solvable in the DDCS; thus, the number of unknowns is dramatically reduced, and the 12 unknown CFMs can be represented by *a* and *k*. Although it is still necessary to establish additional objective functions or complementary equations to solve the CFMs, fewer unknowns are very beneficial to reducing the difficulty of establishing, solving, and optimizing these functions or equations. This is the main contribution of the DDCS.

### 4.2. Minimum Energy Hypothesis to Optimize CFMs

The possibilities of CFMs and joint moments values are not unique even if under the same gait posture. Actively changing the activation of muscles also affects the CFMs and joint moments. In Section 4.1.1, the CFMs *F* are represented as functions of *a* and *k*. In this section, we propose a method to optimize the values of *a* and *k* via a minimum energy hypothesis.

#### 4.2.1. Minimum Energy Hypothesis

The CFMs and joint torques during human walking have been the result of long-term optimization ever since humans learned to walk. Humans prefer gaits and joint torques that are the most comfortable and consume the least energy. It was verified by many experiments that people prefer to move in ways that minimize their energetic cost [37]. The energetic cost of human in locomotion is produced by the exertion of muscles, which are nearly proportional to the joint moments. Therefore, we can approximately quantify the body’s total energy cost by setting up a function of the joint moments. By minimizing the energy function, we can optimize the CFMs in locomotions.

#### 4.2.2. Energy Equations of Human Bodies

According to the minimum energy hypothesis, we can optimize the unknowns *a* and *k* by minimizing an energy function about *a* and *k*. Finally, we choose the form of the energy function as:

(34) $$E=\sum_{i=1}^{n}f_{i}T_{i}^{2}$$

where *E* represents the energy of the wearer, *n* is the number of active joints, and $f_{i}$ is the weight coefficient of joint *i*. It is difficult to establish a digital and precise optimization target to quantize the energy cost of human bodies due to the complexity of human locomotion mechanisms. Therefore, Equation (34) is an approximate evaluation of the instantaneous energy cost of human bodies. For the rationality of Equation (34), we can give two explanations.

**Figure 5:**
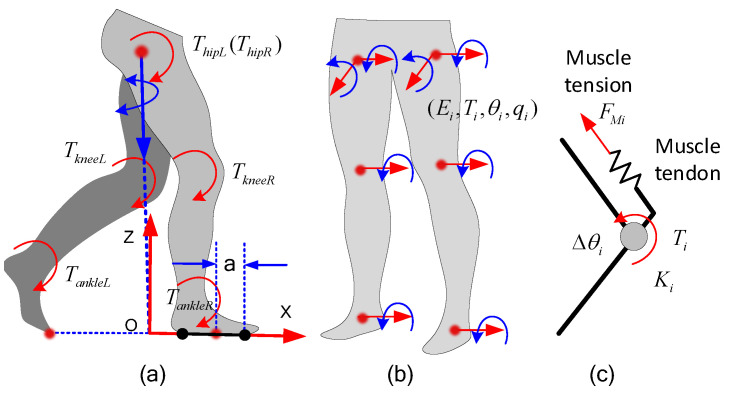
Establishment of energy objective function. (**a**) The positions of COPs are not exclusive: even the joint angles have not changed. The joint moments and COP positions can influence each other. (**b**) Main joints during walking. (**c**) Elastic joint model.

***The elastic potential energy of human joints:*** By calculating the elastic potential energy of human joints, we can derive the energy cost function with the form of Equation (34). As shown in Figure 5c, human joints rotate under the pull of muscle tendons in locomotion, and the pulling forces are provided by the muscle tensions. Muscle tendons are elastic, and thus can be regarded as springs. Therefore, the relationship among the joint moment $T_{i}$, the joint stiffness $K_{i}$, and the deformation quantity of the joint $\Delta \theta_{i}$ is as follows:

(35) $$T_{i}=-K_{i}\Delta \theta_{i}$$

The potential energy function of the joints is:

(36) $$E=\sum_{i=1}^{n}\frac{T_{i}^{2}}{2K_{i}}$$

where $K_{i}$ is the stiffness of joint *i*. It is obvious that by collating, Equation (36) can be written in the form of Equation (34).

***Solving hyperstatic problems in structural engineering:*** In structural engineering, to consider hyperstatic structures, engineers have developed complementary energy methods to establish compatibility equations. By minimizing the complementary energy, hyperstatic problems can be solved. Human bodies can be considered as hyperstatic structures in double-supporting phases [29]. Inspired by the minimum complementary energy principle, we can establish an energy function by calculating the total elastic potential energy of human joints. The function is the same as in Equation (36).

***Energy cost function:*** According to the analysis above, no matter which of the two explanations is chosen, the energy function can be written as shown in Equation (34). Furthermore, according to classical robot dynamics, joint moments can be calculated as:

(37) $$M\left(\theta \right)\ddot{\theta }+C\left(\theta ,\dot{\theta }\right)\dot{\theta }+G\left(\theta \right)+F_{0}=T$$

(38) $$F_{0}=R^{-1}F\;$$

With the assumption that no energy is dissipated in the ground contacts (e.g., via sliding friction) and with the following definitions:

• $\theta ,\dot{\theta },\ddot{\theta }\in$$R^{r}$: the state vector, representing the angle, angular velocity, and angular acceleration of human joints.
• *M*, C∈$R^{r\times r}$, G∈$R^{r}$: the inertia matrix, the Coriolis and centrifugal matrix, and the gravitational vector.
• $F_{0}\in$$R^{r}$: the CFMs under the foot in coordinate system ($O_{0}$–$X_{0}Y_{0}Z_{0}$).
• T∈$R^{n}$: the joint moments.

According to Equations (32), (34), (37), and (38), we can calculate the total potential energy of the joints, which is an explicit function of *a* and *k*.

(39) $$E=f_{E}\left(a,k\right)$$

By optimizing *a* and *k*, we can obtain the minimum of *E*, and the CFMs in these conditions can be calculated using Equation (32).

#### 4.2.3. Optimization to Minimize the Potential Energy

By minimizing the potential energy function of the human body, we can find the corresponding values of *a*, *k*, and, furthermore, the CFMs. The variable *a* represents the distance between COPs and point *A*, as shown in Figure 3. Thus,

(40) $$\left\{\begin{array}{c} 0\leq a\leq l_{f}\\ 0\leq f_{b}\left(a,\theta \right)\leq l_{f} \end{array}\right.$$

where $l_{f}$ is the length of the foot; $f_{b}$ is the relationship between *b* and *a*. *E* is a bivariate quadratic function of *a* and *k*. If one of the variables is a fixed value, we can directly obtain the minimum value of the function. Therefore, we can find the minimum value of *E* using a traversal method, as follows:

• According to Equation (40), we can determine the value range of *a*.
  (41) $$a\in [a_{1},a_{2}]$$
• Assuming that the value of *a* is known, *E* is a unary quadratic equation of *k*, as follows:
  (42) $$E=e_{0}k^{2}+e_{1}k+e_{2}$$
  Since $K_{i}$ in Equation (36) is a positive number, it is obvious that $e_{0}$ in Equation (42) is also a positive number. Thus, we can directly obtain the minimum value $E_{0}$ as follows:
  (43) $$k=-\frac{e_{1}}{2e_{0}}$$
• Then, we can calculate the minimum value $E_{i}$.
  (44) $$E_{i}=\frac{4e_{0}e_{2}-e_{1}^{2}}{4e_{0}}$$
• By traversing *a* between a1 and a2, finally, we obtain all the minimum values $E_{1},\;E_{2},\;...,\;E_{i}$ for each value of $a_{i}$.
• By comparing the value of $E_{1},\;E_{2},\;...,\;E_{i}$, we can find the minimum value of *E* and the corresponding *a* and *k*.

By adjusting the size of the increase in *a*, we can change the final accuracies of *a* and *k*. This traversal method can effectively avoid the problem of falling into a local optimum. Finally, the CFMs can be calculated by Equation (32), and the joint moments can be calculated by Equation (37).

The final process of estimating the CFMs and the joint moments is stated in Algorithm 1:
**Algorithm 1:** Estimation of CFMs and joint moments.
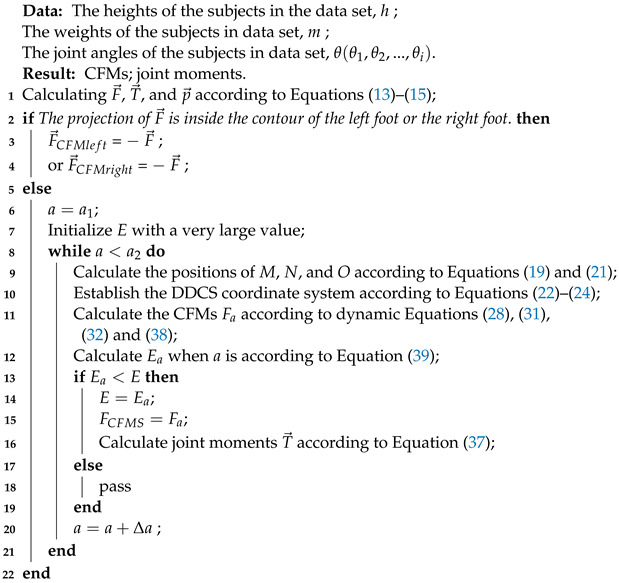


#### 4.2.4. Optimizing the Joint Stiffness

To establish Equation (36), we need the joint stiffness $K_{i}$ of human bodies. The joint stiffness reflects the characteristics of human muscles, cartilage, tendons, and other soft tissues; thus, it is difficult to measure and estimate. In this section, we adopt a traverse method to optimize the values of the $K_{i}$ based on a public data set [38] about human locomotion. In the data set, the researchers measured the human motion data of the subjects with a motion capture system. The CFMs under the subjects’ feet are measured by a dynamic force measuring platform. The motion data includes the angles, the angular velocity, and the angular acceleration of the hip joints, the knee joints, and the ankle joints of the subjects. Then, the joint moments are calculated based on the motion data and the CFMs of the subjects. According to the CFMs and the relative position between the force-measuring platform and the human bodies, $\vec{F}$ and $\vec{T}$ can be calculated.

Since the potential energy of each joint is inversely proportional to the equivalent stiffness of the joint, we just need to determine the ratio of the equivalent stiffness of the different joints. Therefore, we assume the stiffnesses of the flexion/extension of the hip joint is 1, the stiffnesses of the abduction/adduction of the hip joint is $q_{0}$, and the stiffnesses of the flexion/extension of the knee joint and the ankle joint are $q_{1}$ and $q_{2}$. Based on the data in public data sets and Algorithm 1, we can calculate the CFMs and the joint moments $\vec{T}$ if the stiffness of the $\left[q_{0},q_{1},q_{2}\right]$ is certain. We need to find a set of $\left[q_{0},q_{1},q_{2}\right]$ that can minimize the differences in the CFMs between the real values in the data set [38] and the results calculated with Algorithm 1. In this way, the joint factors $\left[q_{0},q_{1},q_{2}\right]$ can be ensured.

## 5. Experimental Verification

### 5.1. Human Simulation Model and Dynamic Parameters

**Figure 6:**
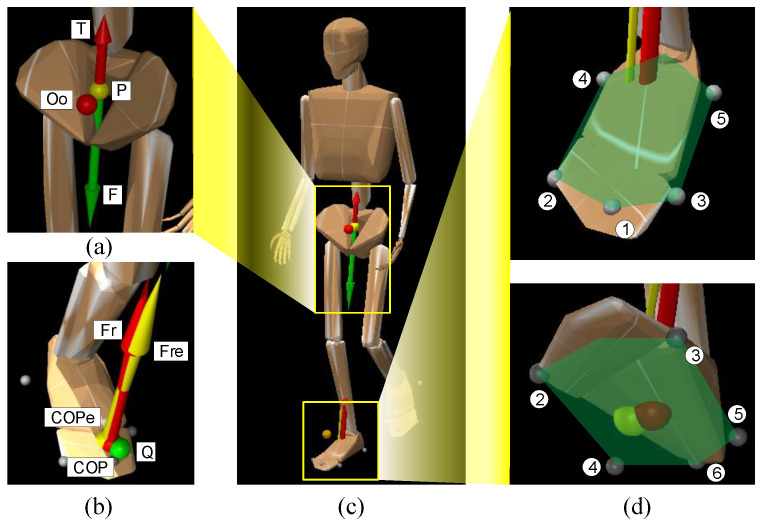
Simulation of human dynamics. (**a**) The resultant inertia force *F* and inertia moment *T*. (**b**) The estimated COPs and CFMs. The red force and red points represent the ground truth, while the green point and yellow force represent the estimated values. (**c**) The human model. (**d**) The six points limit the range of the foot.

In order to calculate the gravities and inertial forces of different human body parts, we adopted an existing human simulation model, as shown in Figure 6. The human simulation model considers the mass and inertia information of different human body parts, and it also includes most of the joints of human bodies. Since the data set was measured in conditions without an exoskeleton, in this experiment, we did not consider the weight of the exoskeleton and treated the human simulation model as a human exoskeleton system. The human simulation model can be invoked by most simulation software that includes a physical engine. Here, we adopted “Mujoco” simulation software, which is an advanced simulator for multi-body dynamics with contact. It is a general-purpose physics engine that aims to facilitate research and development in robotics, biomechanics, graphics and animation, machine learning, and other areas that demand fast and accurate simulation of articulated structures interacting with their environment [39].

By inputting the joint angle of human bodies into the simulation software, the combined inertia force *F* and moment *T* of inertia of the human bodies were calculated, as shown in Figure 6a. To limit the range of the COPs under each foot, we set six points on the plane of each foot, as shown in Figure 6d. According to the range, we could ensure whether *F* projected into the range of one foot and the value range of *a* in Equation (42).

### 5.2. Optimizing $\left[q_{0},q_{1},q_{2}\right]$ Based on a Human Locomotion Dataset

The combination of $\left[q_{0},q_{1},q_{2}\right]$ is a group of hyperparameters, which is supposed to be ensured before utilizing the proposed CFMs estimation method. In this section, we optimize the value of $\left[q_{0},q_{1},q_{2}\right]$ via a data-driven method based on a human locomotion data set [40,41]. The data set collects the height, weight, joint angles, CFMs, and the calculated joint moments of the participants and contains a substantial amount of data. The joint angles were measured using an optical dynamic capture system, and the CFMs were measured with a plane force platform system. According to Algorithm 1, for every group of joint angle inputs, if $\left[q_{0},q_{1},q_{2}\right]$ is ensured, we can acquire the results of the CFMs. Therefore, by finding a set of parameters about $\left[q_{0},q_{1},q_{2}\right]$ that can make the calculated CFMs as close as possible to the real CFMs, $\left[q_{0},q_{1},q_{2}\right]$ can be ensured.

Finally, we choose partial samples from the data set and traverse $q_{1}$ and $q_{2}$ to find a better combination of $\left[q_{0},q_{1},q_{2}\right]$ that makes the optimized CFMs closer to the ground truth. The results of $\left[q_{0},q_{1},q_{2}\right]$ were necessary for estimating the CFMs in the next experiments. The feasible region of $q_{1}$ and $q_{2}$ is (0,5], and the traversing interval is 0.1. Finally, the best combination of $\left[q_{0},q_{1},q_{2}\right]$ on the selected data is:

(45) $$\left[q_{0},q_{1},q_{2}\right]=\left[1.0,\;2.1,\;0.1\right]$$

The result of $\left[q_{0},q_{1},q_{2}\right]$ shows that if the energy of knee joints during walking is appreciated more and the energy of ankle joints is appreciated less, the optimized CFMs are be closer to the ground truth. According to Equation (36), the joint factor is inversely proportional to the joint stiffness. Compared with knee joints, ankle joints have shorter moment arms, smaller slewing ranges, and much stronger tendons; thus, they have higher joint stiffness. This might be a reasonable reason for why the optimized $q_{2}$ is much smaller than $q_{1}$.

### 5.3. Verification of the Proposed CFM Estimation Method

**Figure 7:**
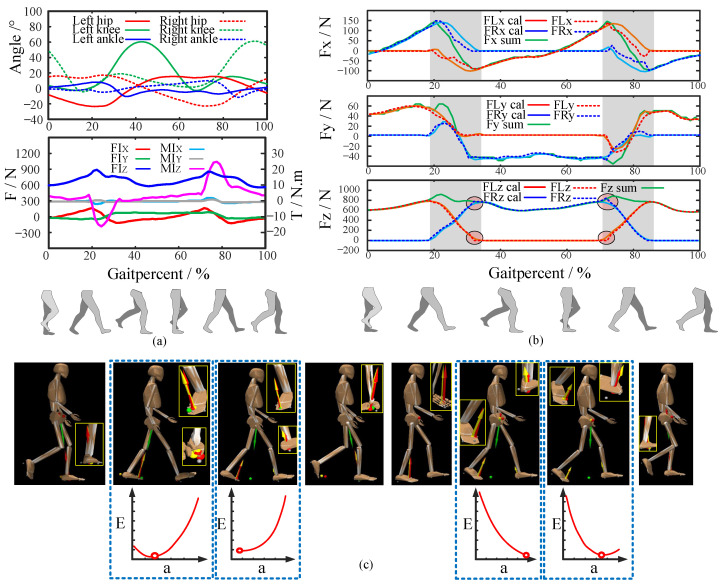
Experiment results. (**a**) The joint angles and resultant inertia force/moments. (**b**) The estimated CFMs. (**c**) CFMs and *E* for some key postures during walking.

To finally verify the performance of the proposed CFM estimation algorithm, we selected a public data set [42] regarding human walking provided by “OpenSim” [43], which is professional human kinematics simulation software. The public data included the joint angles of the participants detected using optical motion capture during walking and a corresponding human body model. The locomotion data and model are pre-calibrated in the geometric and physical dimensions via a residual reduction algorithm [44] provided by “Opensim”, which can be run several times to iteratively generate a model and joint kinematics that together reduce the dynamic inconsistency between measured kinematic and kinetic data. The pre-calibrated locomotion data are limited in amount but high in quality, thus could be used to evaluate the performance of the proposed method. This helped to reduce the deviation caused by mismatches in the mannequin parameters. The joint angles are shown in Figure 7a. Finally, the calibrated model was changed into an available format for “Mujoco” using an open-source tool [45]. Utilizing the calibrated model, locomotion data, and “Mujoco”, the CFMs were estimated via Algorithm 1.

According to Algorithm 1 and the optimized $\left[q_{0},q_{1},q_{2}\right]$ in Equation (45), the CFMs under the foot could be estimated, and the results are shown in Figure 7b. The estimated results were highly similar to the ground truth throughout the whole gait cycle. The calculation errors of $FL_{x}$, $FL_{y}$, $FL_{Z}$, $FR_{x}$, $FR_{y}$, and $FR_{Z}$ during the whole gait cycle were 14.96 ± 28.17 N, 8.05 ± 14.09 N, 11.30 ± 21.89 N, 15.50 ± 28.84 N, 9.20 ± 13.39 N, and 12.27 ± 22.99 N, respectively. The calculation errors of FL and FR were 10.04 ± 21.19 N and 14.14 ± 26.25 N. These results highlight the precision of the proposed method.

However, as marked in Figure 7b, when switching between the single-support state and the double-support state, the calculation deviations of $F_{Z}$ become a little more obvious. These deviations might be caused by the measurement deviations of the joint angles. When these measurement deviations appear, the subpoint of *F* enters or leaves the range of one foot earlier or later. The misjudgment of the support state is the main cause of these deviations marked in Figure 7b. Furthermore, Figure 7c shows the estimated CFM results and the change in *E* with *a* for some key postures. According to the results, in the double-support state, if the COP of the front foot approaches the ball of the front foot and the COP of the back foot approaches the back heel, the energy *E* reaches its minimum value. This matches with practical common sense, thus confirming the rationality of the energy function Equation (39).

## 6. Conclusions

In this paper, we proposed a novel method for estimating the CFMs based on a DDCS and a minimum energy hypothesis. The former transforms the contact forces and moments into five solvable components and two parameters to be optimized, while the latter provides a method to solve the two parameters. The method reduces the number of contact force and moment components that exceed the constraint equations. We verified the accuracy of this method using a publicly available human walking data set. The validation showed that the proposed method is able to estimate the contact forces and moments sufficiently well. This study provides an effective method to calculate the CFMs under the feet, thus contributing to reducing the research and development costs of exoskeletons by overcoming the need to use expensive plantar sensors. The sensor-free approach also reduces the dependency on high-precision, portable, and comfortable CFM detection sensors, which are usually difficult to design.

There are also some limitations of the proposed method. First, to simplify the math and the dynamic model, we ignore the moment component $M_{z}$ in DDCS and assume the COPs are always on lines AB and CD. This reduces the difficulty of estimating the CFMs but also minimally influences the precision and adaptability. Second, the method was developed to estimate the CFMs of human bodies under natural locomotion. If the participant deliberately changes the COPs via inward/lateral weight shifting without any changes in posture, the proposed method cannot accurately estimate the changed CFMs.

## Data Availability

Data are contained within the article.

## Abbreviations

The following abbreviations are used in this manuscript:

DDCS	Dynamic decoupled coordinate system
CFMs	Contact forces and moments
COPs	Centers of pressure

## References

[1] Ding Y., Kim M., Kuindersma S., Walsh C.J. (2018). Human-in-the-loop optimization of hip assistance with a soft exosuit during walking. Sci. Robot..

[2] Kim J., Lee G., Heimgartner R., Revi D.A., Karavas N., Nathanson D., Galiana I., Eckert-Erdheim A., Murphy P., Perry D. (2019). Reducing the metabolic rate of walking and running with a versatile, portable exosuit. Science.

[3] Gordon D.F., McGreavy C., Christou A., Vijayakumar S. (2022). Human-in-the-Loop Optimization of Exoskeleton Assistance Via Online Simulation of Metabolic Cost. IEEE Trans. Robot..

[4] Lee Y., Lee J., Choi B., Lee M., Roh S.G., Kim K., Seo K., Kim Y.J., Shim Y. (2019). Flexible gait enhancing mechatronics system for lower limb assistance (GEMS L-type). IEEE/ASME Trans. Mechatron..

[5] Seo K., Lee J., Park Y.J. (2017). Autonomous hip exoskeleton saves metabolic cost of walking uphill. Proceedings of the 2017 International Conference on Rehabilitation Robotics (ICORR).

[6] Vu H.T.T., Gomez F., Cherelle P., Lefeber D., Nowé A., Vanderborght B. (2018). ED-FNN: A new deep learning algorithm to detect percentage of the gait cycle for powered prostheses. Sensors.

[7] Kang I., Molinaro D.D., Duggal S., Chen Y., Kunapuli P., Young A.J. (2021). Real-time gait phase estimation for robotic hip exoskeleton control during multimodal locomotion. IEEE Robot. Autom. Lett..

[8] Wu X., Ma Y., Yong X., Wang C., He Y., Li N. (2019). Locomotion mode identification and gait phase estimation for exoskeletons during continuous multilocomotion tasks. IEEE Trans. Cogn. Dev. Syst..

[9] Fournier B.N., Lemaire E.D., Smith A.J., Doumit M. (2018). Modeling and simulation of a lower extremity powered exoskeleton. IEEE Trans. Neural Syst. Rehabil. Eng..

[10] Karatsidis A., Bellusci G., Schepers H.M., De Zee M., Andersen M.S., Veltink P.H. (2016). Estimation of ground reaction forces and moments during gait using only inertial motion capture. Sensors.

[11] Mahdavian M., Arzanpour S., Park E.J. (2019). Motion generation of a wearable hip exoskeleton robot using machine learning-based estimation of ground reaction forces and moments. Proceedings of the 2019 IEEE/ASME International Conference on Advanced Intelligent Mechatronics (AIM).

[12] Lee S., Jang J., Park S. (2013). Control of a lower limb exoskeleton using GRF estimation. Proceedings of the IEEE ISR 2013.

[13] Peng Y., Zhang Z., Gao Y., Chen Z., Xin H., Zhang Q., Fan X., Jin Z. (2018). Concurrent prediction of ground reaction forces and moments and tibiofemoral contact forces during walking using musculoskeletal modelling. Med. Eng. Phys..

[14] Alves S.A., Polzehl J., Brisson N.M., Bender A., Agres A.N., Damm P., Duda G.N. (2022). Ground reaction forces and external hip joint moments predict in vivo hip contact forces during gait. J. Biomech..

[15] Hossain M.S.B., Guo Z., Choi H. (2023). Estimation of Lower Extremity Joint Moments and 3D Ground Reaction Forces Using IMU Sensors in Multiple Walking Conditions: A Deep Learning Approach. IEEE J. Biomed. Health Inform..

[16] Li G., Liu T., Yi J., Wang H., Li J., Inoue Y. (2016). The lower limbs kinematics analysis by wearable sensor shoes. IEEE Sens. J..

[17] Tedesco S., Perez-Valero E., Komaris D.S., Jordan L., Barton J., Hennessy L., O’Flynn B. (2020). Wearable motion sensors and artificial neural network for the estimation of vertical ground reaction forces in running. Proceedings of the 2020 IEEE SENSORS.

[18] Willemstein N., Sridar S., van der Kooij H., Sadeghi A. (2023). 3D Printed Graded Porous Sensors for Soft Sensorized Insoles with Gait Phase & Ground Reaction Forces Estimation. arXiv.

[19] Amici C., Ragni F., Ghidoni M., Fausti D., Bissolotti L., Tiboni M. (2020). Multi-sensor validation approach of an end-effector-based robot for the rehabilitation of the upper and lower limb. Electronics.

[20] Choi H.S., Shim M., Lee C.H., Baek Y.S. (2018). Estimating grf (ground reaction force) and calibrating cop (center of pressure) of an insole measured by an low-cost sensor with neural network. Proceedings of the 2018 IEEE 18th International Conference on Bioinformatics and Bioengineering (BIBE).

[21] Wang D., Cai P., Mao Z. (2016). The configuration of plantar pressure sensing cells for wearable measurement of COP coordinates. Biomed. Eng. Online.

[22] Wu H., Ma H., Wei Q., Wang J., An H. (2018). A novel force control method of lower limb exoskeleton based on dual foot force sensors. Proceedings of the 2018 Chinese Control And Decision Conference (CCDC).

[23] Fei C., Xu D., Wang Z., Wang B. (2017). Research on the exoskeleton robot foot pressure detection system based on curve fitting. Proceedings of the 2017 IEEE 3rd Information Technology and Mechatronics Engineering Conference (ITOEC).

[24] Nabipour M., Moosavian S.A.A. (2018). Dynamics modeling and performance analysis of RoboWalk. Proceedings of the 2018 6th RSI International Conference on Robotics and Mechatronics (IcRoM).

[25] Lancini M., Serpelloni M., Pasinetti S., Guanziroli E. (2016). Healthcare sensor system exploiting instrumented crutches for force measurement during assisted gait of exoskeleton users. IEEE Sens. J..

[26] Ryu H.X., Park S. (2018). Estimation of unmeasured ground reaction force data based on the oscillatory characteristics of the center of mass during human walking. J. Biomech..

[27] Azimi V., Nguyen T.T., Sharifi M., Fakoorian S.A., Simon D. (2018). Robust ground reaction force estimation and control of lower-limb prostheses: Theory and simulation. IEEE Trans. Syst. Man Cybern. Syst..

[28] Kazerooni H., Racine J.L., Huang L., Steger R. (2005). On the control of the berkeley lower extremity exoskeleton (BLEEX). Proceedings of the 2005 IEEE International Conference on Robotics and Automation.

[29] Vantilt J., Giraddi C., Aertbeliën E., De Groote F., De Schutter J. (2018). Estimating contact forces and moments for walking robots and exoskeletons using complementary energy methods. IEEE Robot. Autom. Lett..

[30] Kloeckner J., Visscher R., Taylor W.R., Viehweger E., De Pieri E. (2023). Prediction of ground reaction forces and moments during walking in children with cerebral palsy. Front. Hum. Neurosci..

[31] Oh J., Kuenze C., Signorile J.F., Andersen M.S., Letter M., Best T.M., Ripic Z., Emerson C., Eltoukhy M. (2021). Estimation of ground reaction forces during stair climbing in patients with ACL reconstruction using a depth sensor-driven musculoskeletal model. Gait Posture.

[32] Kazerooni H., Steger R., Huang L. (2006). Hybrid control of the Berkeley lower extremity exoskeleton (BLEEX). Int. J. Robot. Res..

[33] Ghan J., Steger R., Kazerooni H. (2006). Control and system identification for the Berkeley lower extremity exoskeleton (BLEEX). Adv. Robot..

[34] Kumar V., Hote Y.V., Jain S. (2019). Review of exoskeleton: History, design and control. Proceedings of the 2019 3rd International Conference on Recent Developments in Control, Automation & Power Engineering (RDCAPE).

[35] Sun Y., Tang Y., Zheng J., Dong D., Chen X., Bai L. (2022). From sensing to control of lower limb exoskeleton: A systematic review. Annu. Rev. Control.

[36] Yi X., Zhou Y., Habermann M., Shimada S., Golyanik V., Theobalt C., Xu F. Physical inertial poser (pip): Physics-aware real-time human motion tracking from sparse inertial sensors. Proceedings of the IEEE/CVF Conference on Computer Vision and Pattern Recognition.

[37] Selinger J.C., O’Connor S.M., Wong J.D., Donelan J.M. (2015). Humans can continuously optimize energetic cost during walking. Curr. Biol..

[38] Camargo J., Ramanathan A., Flanagan W., Young A. (2021). A comprehensive, open-source dataset of lower limb biomechanics in multiple conditions of stairs, ramps, and level-ground ambulation and transitions. J. Biomech..

[39] Limited Liability Company, Roboti. MuJoCo. https://mujoco.readthedocs.io/en/stable/overview.html.

[40] Wojtusch J., von Stryk O. (2015). Humod-a versatile and open database for the investigation, modeling and simulation of human motion dynamics on actuation level. Proceedings of the 2015 IEEE-RAS 15th International Conference on Humanoid Robots (Humanoids).

[41] HuMoD. https://www.informatik.tu-darmstadt.de/sim/forschung_sim/datensaetze_sim/humod_sim/index.en.jsp.

[42] Simtk. https://simtk.org/frs/?group_id=773.

[43] OpenSim. https://opensim.stanford.edu/.

[44] Delp S.L., Anderson F.C., Arnold A.S., Loan P., Habib A., John C.T., Guendelman E., Thelen D.G. (2007). OpenSim: Open-source software to create and analyze dynamic simulations of movement. IEEE Trans. Biomed. Eng..

[45] O2MConverter. https://github.com/aikkala/O2MConverter.

